# Liquid Crystalline Networks Hamper the Malignancy of Cancer Cells

**DOI:** 10.1002/adhm.202403607

**Published:** 2025-01-19

**Authors:** Daniele Martella, Ignazia Tusa, Alessandro Tubita, Alessia Negri, Marco Turriani, Marta Rojas‐Rodríguez, Martina Salzano de Luna, Alessio Menconi, Camilla Parmeggiani, Elisabetta Rovida

**Affiliations:** ^1^ Department of Chemistry “Ugo Schiff” University of Florence via della Lastruccia 3–13 Sesto Fiorentino 50019 Italy; ^2^ European Laboratory for Non Linear Spectroscopy (LENS) via N. Carrara 1 Sesto Fiorentino 50019 Italy; ^3^ Department of Clinical and Experimental Biomedical Sciences University of Florence Viale G.B. Morgagni, 50 Florence 50134 Italy; ^4^ Department of Chemical, Materials and Industrial Production Engineering University of Naples Federico II Piazzale V. Tecchio, 80 Napoli 80125 Italy

**Keywords:** A375 melanoma cells, cancer, cellular senescence, liquid crystalline networks, photopolymerizations, polymeric cell scaffolds, tumor aggressiveness

## Abstract

Mimicking compositions and structures of extracellular matrix is widely studied to create in vitro tumor models, to deepen the understanding of the pathogenesis of the different types of cancer, and to identify new therapies. On the other hand, the use of synthetic materials to modulate cancer cell biology and, possibly, to reduce the malignancy of cancer cells through their exploitation is far less explored. Here, the study evaluates the effects of Liquid Crystalline Networks (LCNs) based scaffolds on the growth of A375 metastatic melanoma cells. Interestingly, cells grown on such materials show reduced cell proliferation and colony‐forming capacity with respect to those cultivated on standard plates. These effects are associated with a higher percentage of senescent cells and a shift to a more epithelial phenotype, pointing to the occurrence of a mesenchymal to epithelial transition. All these biological outcomes are affected by the amount of crosslinker in the material and have been induced only thanks to the interactions with the polymeric substrate without the need of further chemical (e.g., specific growth factor) or physical (e.g., irradiation) stimuli, opening to the possible development of anti‐cancer coatings.

## Graphical abstract

Liquid Crystalline Networks are used as scaffolds for the growth of A375 melanoma cells demonstrating to affect their malignancy. Indeed, only by contact, these materials reduces cell proliferation and colony formation capacity, while increasing the number of senescent cells and promoting the mesenchymal to epithelial transition.

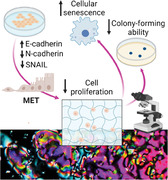

## 1 Introduction

Cancer cell phenotype is strongly dependent on its microenvironment, and extracellular matrix (ECM) surrounding tumors is generally characterized by an abnormal remodeling, both regarding its composition and structure.^[^ ^1^ ^]^ Indeed, the interactions between cancer cells and ECM have a dominant role in regulating the formation, progression, and spreading of tumors, and a primary target of tissue engineering has been on trying to reproduce such interactions in vitro. Mimicking the ECM with biomaterials is a fundamental objective of current research to create physiological systems that resemble tissue structure,^[^ ^2^ ^]^ to deepen the biological basis of cancer and to test novel therapeutic approaches. For this reason, a plethora of natural and synthetic materials have been tested to mimic as much as possible the structure of native tissues.^[^ ^1^, ^3^ ^]^ On the contrary, the use of materials able per se to affect the malignancy of cancer cells upon contact and to improve or to integrate the therapeutic benefits of other strategies has been scarcely explored. As an example, the antineoplastic effect of synthetic materials might be exploited for the treatment of melanoma, a malignant tumor that arises following the neoplastic transformation of melanocytes, located in cutaneous or extracutaneous sites, with a constantly growing incidence in the population.^[^ ^4^ ^]^ The optimal therapeutic approach in early stages is surgical resection, while for patients with metastatic melanoma available treatments have greatly improved the survival but are still unsatisfactory.^[^ ^4^ ^]^ Moreover, not all melanomas can be (completely) removed due to their extension, position, proximity to organs and vessels, and prosthetic rehabilitation might be needed in some cases, including after the enucleation of the eye or the partial maxillectomy in case of uveal or palate melanoma, respectively, so that new solutions are required. In this scenario, the identification of materials endowed with antineoplastic effects would be of extreme interest to reduce tumor malignancy, to prevent tumor recurrence, and/or to be used for prosthetic rehabilitation.^[^ ^5^ ^]^ This would be possible by the development of scaffolds able to promote the mesenchymal to epithelial transition (MET), a reversible biological process that involves the transition from mesenchymal cells (that are motile, spindle‐shaped cells endowed with invasive ability) to planar arrays of epithelial ones. The presence of the former phenotype is associated with metastatic progression and MET can limit this process. However, only scaffolds that are able to promote the opposite epithelial to mesenchymal transitions (EMT)^[^ ^6^ ^]^ or cellular senescence have been described to develop new tumor models and to evaluate how 3D morphology and mechanical properties affect the cell response. Among recent examples, poly(ε‐caprolactone) (PCL) based electrospun fibers showing mesh‐like structures were used in the presence of a hepatocyte growth factor (EMT inducer) leading to both epithelial and mesenchymal phenotypes of Madin‐Darby Canine Kidney cells, depending on the fiber diameters.^[^ ^7^ ^]^ In another study, breast cancer cells cultured in the presence of transforming growth factor β on PCL based scaffolds showed the dependence of EMT from the fiber organization.^[^ ^8^ ^]^ 3D porous chitosan based scaffolds were demonstrated more effectively for prostate cancer EMT than 2D one,^[^ ^9^ ^]^ while polymer elasticity was demonstrated decisive to maintain the epithelial phenotype of MCF‐7 cells (a breast cancer cell line) on poly(caprolactone‐co‐D,L‐lactide) scaffolds.^[^ ^10^ ^]^

Regarding cellular senescence, the process leads to a stable cell cycle arrest limiting cancer progression, and can be induced by a variety of stimuli such as oxidative stress, telomere shortening, DNA damage or certain oncogenes.^[^ ^11^ ^]^ Only few reports described materials able to induce senescence and, between them, poly(ε‐caprolactone‐co‐D,L‐lactide) scaffolds have been demonstrated for lung carcinoma cells, highlighting the importance of fine tuning the material elasticity.^[^ ^12^ ^]^ Furthermore, in PCL scaffolds with specific irradiation (to start senescence) were demonstrated the importance of the 3D structuration in the senescence process.^[^ ^13^ ^]^

To date, and to the best of our knowledge, all the reported scaffolds are used to reinforce or suppress the effect of other chemical (e.g., growth factor) or physical (e.g., irradiation) stimuli, while remain unexplored the ability of bare materials to affect a specific antitumoral cellular response. In this scenario, with the aim to engineer materials able themselves to induce specific responses in tumoral cells, we present here a study on the use of Liquid Crystalline Networks (LCNs) based scaffolds for culturing A375 melanoma cells. LCNs are liquid crystalline polymers, offering a unique combination between mechanical properties of crosslinked networks and the ability of liquid crystals to self‐organize into anisotropic phases.^[^ ^14^ ^]^ Interestingly, such materials found application in different research fields,^[^ ^15^ ^]^ and have been already studied as cellular scaffolds^[^ ^16^ ^]^ to demonstrate their ability to implement myotube formation,^[^ ^17^ ^]^ to support the aligned growth of different cell lines (e.g., myotubes and fibroblasts)^[^ ^18^ ^]^ and to implement the maturation of human induced pluripotent stem cell‐derived cardiomyocytes.^[^ ^19^ ^]^

Such results highlighted how material composition was a key parameter to implement the cell growth, and we decided to explore the ability of LCNs of different stiffness to induce specific responses in malignant tumor cells toward the development of anti‐cancer materials. Therefore, we report here on the evaluation of LCNs of different stiffness, and on their ability to reduce melanoma A375 cell line proliferation when used as cellular scaffolds. Interestingly, a reduction in colony‐forming ability as well as an increase in cellular senescence were demonstrated together with the ability of the scaffold to promote the shift from a mesenchymal to a more epithelial phenotype. An important highlight is that both MET and senescence processes are induced by the material without any other supply of growth factors or irradiations.

## 2 Results and Discussion

### 2.1 Scaffold Preparation and Characterization

**Figure 1:**
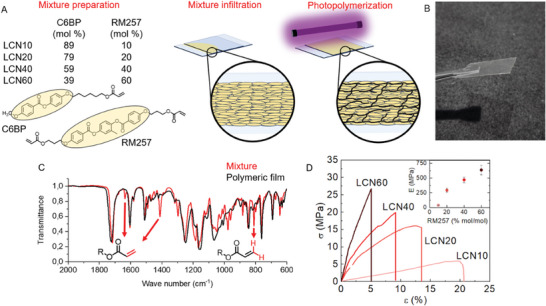
Scaffold preparation and characterization. A) Composition of the monomer mixture and main steps of the film fabrication. The mixture was infiltered in a polymerization cell where the monomers were able to self‐assemble in a homogeneous planar monodomain that is stabilized by UV photopolymerization. B) Optical image of the final free‐standing polymeric thin films with good transparency. C) Comparison of ATR‐IR spectra of a monomeric mixture and a final polymerized material (shown for LCN40). D) Representative engineering stress‐strain curves of LCNs. Young's modulus of the different formulations is shown in the inset.

LCN scaffolds were prepared through radical photopolymerization of a mixture of commercial reactive mesogens following a standard procedure as reported in **Figure** **1**A.^[^ ^20^ ^]^ The monomeric mixtures contained the mesogen C6BP, the liquid crystal crosslinker RM257, and the radical photoinitiator Irgacure 369. The relative amounts of C6BP (from 89% mol/mol to 39% mol/mol) and RM257 (from 10% mol/mol to 60% mol/mol) were varied to obtain a set of 4 materials with different physico‐chemical properties. The radical photoinitiator Irgacure 369 (1% mol/mol) was added to control the polymerization with UV light.

The different materials are called in this article as LCNx where x is the percentage of RM257 present in the monomeric mixture (e.g., LCN10 contains 10% mol/mol of crosslinker).

All the mixtures prepared show a liquid crystalline nematic phase according to the observation at the Polarized Optical Microscope (POM) with a clearing point ≈55 °C (see Figure S1, Supporting Information). The mixtures were heated at 65 °C in the isotropic phase and infiltrated into specific polymerization cells. The polymerization cells were composed of two glasses separated by 50 µm‐sized spacers to control the thickness of the final product. The glasses were previously coated with a layer of polyvinyl alcohol (PVA) and rubbed unidirectionally with a velvet cloth to obtain a sacrificial aligning surface.

After the complete infiltration, the sample was cooled down under the clearing point (45 °C) where the mixtures showed the liquid crystalline phase. The mesogens, driven by the aligning surface, were able to self‐assemble in a homogeneous planar monodomain as observed by POM images reported in Figure S2 (Supporting Information). The samples were irradiated for 10 minutes with a 385 nm LED lamp to promote the homolytic dissociation of the radical initiator Irgacure 369 and start the polymerization of the acrylate groups. Then, the samples were further irradiated at 65 °C for 10 minutes to obtain the complete conversion of the monomers.^[^ ^21^ ^]^ The final products were free‐standing polymeric transparent films with a thickness of 50 µm (Figure 1B).

The successful polymerization reaction was monitored through attenuated total reflectance‐infrared (ATR‐IR) spectroscopy, observing the disappearance of characteristic bands of the acrylate group as shown in Figure 1C. In particular, the spectra of the mixtures present characteristic bands (1635 and 1410 cm^−1^ relative to the C ═ C double bond in the acrylate group and a characteristic band relative to the ═ CH_2_ at 811 cm^−1^)^[^ ^22^ ^]^ that disappears in the spectra of the polymerized products.

As already reported,^[^ ^17^, ^18^, ^19^ ^]^ polymeric films prepared with this methodology are bulk materials, not presenting porosity (at least not detectable by atomic force or electron microscopies) nor a nanometric structuring of the surface. An example of the smooth surface of the materials is reported in Figure S3 (Supporting Information) for LCN20. Moreover, the polymer chemical structure determined an hydrophilic characteristics of the scaffold (with water contact angle ≈70° for all the formulations)^[^ ^18a^ ^]^ and (in combination with the absence of porosity) swelling in water or other aqueous formulations (e.g., PBS) was not present unless specific treatments are performed on the standard LCN.^[^ ^23^ ^]^

Indeed, the different mixture compositions mainly affected the mechanical properties of the samples^[^ ^24^ ^]^ that have been characterized by tensile tests at physiological temperature (the same temperature of the following cell cultures). Representative engineering stress‐strain curves of the LCNs are shown in Figure 1D. A significant effect of the crosslinking degree can be recognized. A higher density of covalent links, indeed, hampers the molecular mobility, which reflects in a stiffening of the material at the macroscopic scale. As a result, scaffolds with a higher amount of crosslinker in the formulation are characterized by a higher Young's modulus, can tolerate higher maximum stresses, but are also characterized by a lower deformability. To be more quantitative, the Young's modulus is reported in the inset of Figure 1D as a function of the amount of crosslinker. Apart from a more marked increase between LCN10 and LCN20, the value of E seems to linearly increase with the amount of the crosslinker. A similar trend is also followed by the maximum stress, which steadily grows by increasing the crosslinking degree. Differently, the strain at break significantly reduces, meaning that the stiffening of the samples comes along with their embrittlement. For the sake of clarity, all the mechanical properties extracted from Figure 1D are summarized in **Table** **1**.

**Table 1:** Mechanical properties of the LCNs.

Sample	E [MPa]	σ_max_ [MPa]	ɛ_B_ [%]
LCN10	38.4 ± 9.1	5.6 ± 1.6	20.1 ± 2.9
LCN20	291.9 ± 36.4	14.9 ± 2.1	14.6 ± 2.9
LCN40	468.5 ± 49.2	19.2 ± 2.9	10.3 ± 3.4
LCN60	635.6 ± 81.3	24.3 ± 1.4	6.8 ± 3.5

^*^Standard Petri dishes (TC‐Plate 6 well standard 83.3920) used in the following biological assays as controls present a Young Modulus in the GPa range.

### 2.2 Effects of LCN Scaffolds on the Proliferation and Colony‐Forming Ability of A375 Cells

**Figure 2:**
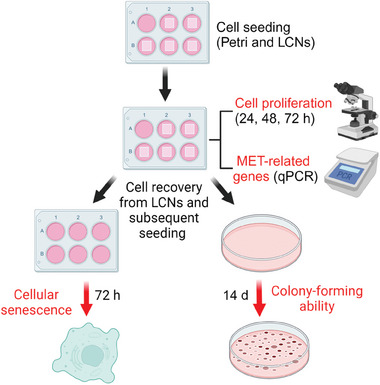
Scheme of the biological experiments performed. Cells were plated in 6‐well multiwell or LCNs and maintained in culture for 72 hours. Pictures were taken after the indicated times to evaluate cell proliferation. Alternatively, cells were lysed and mRNA extracted to perform qPCR to quantify the expression levels of EMT‐related genes. In other experiments, cells were detached from the control plates or the LCNs after 72 hours and then plated on plastics to perform cellular senescence assays after 72 additional hours, or colony forming ability assays after 14 days. Created in BioRender. Tubita, A. (2023) BioRender.com/c00i536.

To investigate the ability of LCN scaffolds to modulate the biological functions of cancer cells, A375 melanoma cells were seeded on LCNs and on standard plates, as control. A comprehensive scheme of the different biological tests performed in this study is reported in **Figure** **2**.

**Figure 3:**
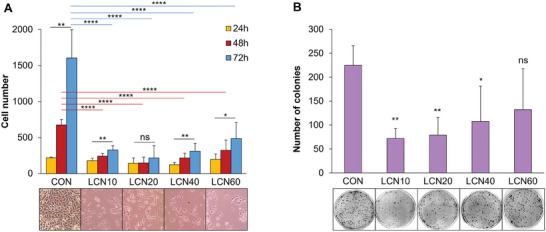
LCN scaffolds markedly reduce the proliferation and colony‐forming ability of A375 melanoma cells. A) A375 melanoma cells were seeded on LCNs scaffolds and on standard plates used as control (CON). Pictures were taken using a microscope with a 10X objective after 24, 48, and 72 hours and the cell number was evaluated using the ImageJ software from 4 random pictures/samples. The data reported represent averages ± SD from five independent experiments. Representative pictures from 72‐hour samples are included. *p ≤ 0.05,  **p ≤ 0.01, and ****p ≤ 0.0001 as determined by Student's *t* test. ns, not significant. B) Cells treated as above described for 72 hours were detached from LCNs and standard plates (CON), seeded at low density and allowed to grow for 14 days. Values are means ± SD of data from four independent experiments. *p ≤ 0.05 and **p ≤ 0.01 as determined by Student's *t* test. ns, not significant. Representative images from each condition are shown.

First, the proliferative capacity of cancer cells on LCNs bearing different crosslinker percentages was analyzed. The experiments highlighted a lower proliferation of cells grown on all types of LCNs if compared to standard plates (**Figure** **3**A); this is even more evident if we consider that a lower number of cells were seeded on the plate with respect to the LCNs (7.5 × 10^4^ and 1.5 × 10^5^, respectively). Melanoma cells were nevertheless able to grow on LCNs supports, as witnessed by the significant increase of the number of cells after 72 hours if compared to those counted after 24 hours on all LCNs, except for LCN20. The reason for the impairment of growth on LCN20 may be due to the increased cellular senescence (see below).

On the contrary, no differences were observed among the number of cells counted after 72 hours on the different LCNs, although a slightly greater proliferation, not reaching significance, was observed on LCN60 if compared to the other LCNs. From the results obtained, we can deduce that the materials tested, irrespectively of the cross‐linker percentage, do not constitute an optimal support for the growth of melanoma cells, rather disfavoring their proliferation, at least if compared to the substrates normally used for cell cultures. This behavior is typical of many other materials that lack cell‐signaling or cell‐attachment points, but in addition, our LCNs demonstrated a very interesting effect on senescence and gene expression, as reported below.

We next evaluated the effects of the different LCNs on the colony‐forming ability of melanoma cells, another important property of malignancy. To this end, after 72 hour‐growth on LCNs or control plates, colony assays were carried out. The results highlight a marked decrease in the ability to form colonies of cells grown on LCNs if compared to cells grown on culture plates (Figure 3B). Interestingly, a marked reduction in the number of colonies was found for cells previously grown on LCNs with a lower percentage of crosslinkers (i.e., LCN10 and LCN20).

Overall, the above results seem to be in line with the notion that tumor cells require specific ECM properties, among them mostly leading to increased stiffness, to undergo an optimal survival and proliferation and therefore to be able to support the growth of the neoplastic mass. The latter, indeed, is supported and strengthened by changes that the ECM undergoes during the development and progression of the neoplastic mass. Along this line, the reduction in the number of colonies formed by cells previously grown on LCNs supports, could be ascribed, among other factors, to their rigidity, not suitable for the ability to form new colonies starting from a single cell.

### 2.3 Effect of LCN Scaffolds on the Senescence of A375 Cells

**Figure 4:**
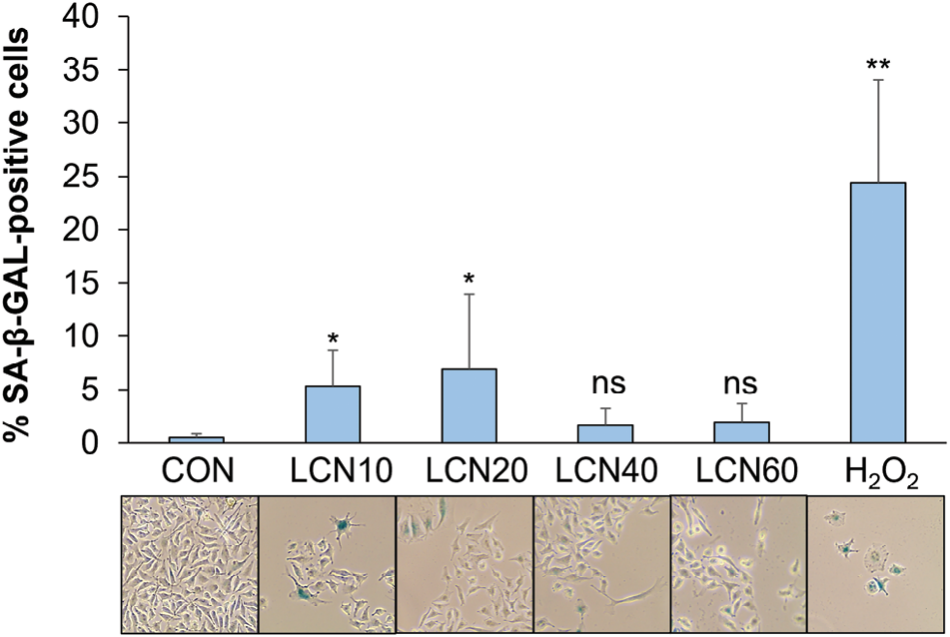
LCN scaffolds increase SA‐βGal positivity in A375 melanoma cells. Cells were seeded on LCNs at different crosslinker percentages and on standard plates (CON, control). After 72 hours, cells were detached and seeded on culture plates for an additional 72 hours. Treatment with 200 µm H_2_O_2_ during the first 2 hours was used as a positive control for SA‐βGalactosidase assay. The percentage of SA‐βGal–positive cells with respect to the total number of cells was calculated from six different 20X magnified fields from four independent experiments using ImageJ software. Values are means ± SD. *p ≤ 0.05, **p ≤ 0.01 versus control (CON) as determined by Student's *t* test. ns, not significant. Representative images from each condition are shown.

Cellular senescence is characterized by an irreversible growth arrest therefore, its occurrence is among the desirable effects of any treatment in cancer. To verify whether LCN scaffolds elicit cellular senescence of melanoma cells, we performed an assay, after 72 hours of growth on different LCNs or on control plates, to quantify the senescence‐associated (SA) β‐galactosidase activity (Figure 2). This is the most common marker of lysosomal activity and one of the first tests originally used to assess senesce in vitro.^[^ ^25^ ^]^ The results reported in **Figure** **4** highlight an increased percentage of senescent cells after growth on LCNs with a lower percentage of crosslinker (LCN10 and LCN20) which is significant if compared to the control. Treatment with H_2_O_2_, a powerful inducer of cellular senescence extensively used as internal control in the SA‐β‐galactosidase assay, was used as a positive control. Given that tumor cells require a suitable ECM to stimulate their growth and the development of their malignant characteristics, it is possible that a support that does not present these necessary properties induces an arrest of the cell cycle in tumor cells and therefore of their ability to proliferate. Indeed, the mechanism by which the cells adhere onto LCNs, whether it depends on integrins and/or on other adhesion molecules, has not been investigated and should be dissected in future studies.

### 2.4 Effect of LCN Scaffolds on the Epithelial versus Mesenchymal Phenotype Transition in A375 Cells

**Figure 5:**
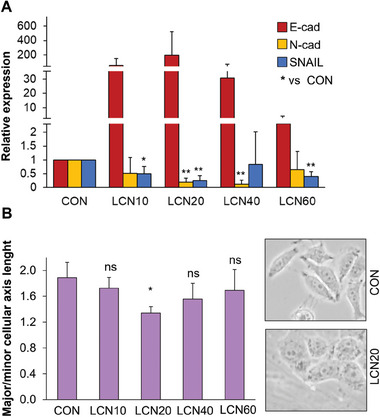
LCN scaffolds induce mesenchymal to epithelial transition in A375 melanoma cells. A) A375 melanoma cells were seeded on LCNs at different crosslinker percentages and on standard plates (CON, control). After 72 hours, cells were lysed and qPCR for the indicated genes was performed. The data were normalized with respect to the mean of GAPDH and 18S mRNA levels and expressed as fold‐change with respect to the values obtained for control samples. The data reported represents the average ± SD from four experiments. *p ≤ 0.05 and **p ≤ 0.01 versus control as determined by Student's *t*‐test. B) Alternatively, after 72 hours pictures were taken, and axis lengths were measured (pixels). The graph shows the major/minor cellular ratios. Data reported represents the average ± SD from three experiments. *p ≤ 0.05 versus control as determined by Student's *t*‐test. ns, not significant. Representative images of control (CON) and LCN20 cultures are reported.

As an additional test to shed light on the possible effects of LCNs on the malignant phenotype of A375 cells, we evaluated the expression of genes associated with epithelial (E‐cadherin) to mesenchymal (N‐cadherin and SNAIL) phenotypes^[^ ^26^ ^]^ via quantitative PCR (qPCR) after 72 hours of growth on LCNs and on culture plates. The obtained data show a robust increase (from one to two orders of magnitude) in the amount of the mRNA for E‐cadherin, a protein associated with the epithelial phenotype, in cells grown on the various types of LCNs if compared to the controls grown on plates (**Figure** **5**A). We can observe how E‐cadherin mRNA is greatly increased in cells grown on LCNs with a lower percentage of crosslinkers, i.e., LCN10 and LCN20. As regards the genes associated with the mesenchymal phenotype, we observed a significant decrease in the expression of N‐cadherin and SNAIL in cells grown on the different types of LCNs if compared with the controls. Importantly, cell shape analysis showed that cells cultured on the different LCNs tend to display a reduced mesenchymal phenotype (i.e., are less elongated as witnessed by the reduced ratio between major and minor cellular axes) than those cultured on the control plastics (Figure 5B). This ratio reached statistical significance in cells cultured on LCN20, further supporting the results shown in Figure 5A. It is known that the rigidity and density of the extracellular matrix favors the EMT transition and the development of the malignant characteristics of tumor cells.^[^ ^27^ ^]^ We can therefore conclude that the tested supports, probably also due to their intrinsic rigidity, do not favor the transition of tumor cells from epithelial to mesenchymal phenotype and, consequently, the aforementioned capabilities.

## 3 Conclusion

This study is focused on the effects of the growth of neoplastic cells on supports made of LCNs with different molecular compositions. Four types of LCN scaffolds have been prepared with an increasing percentage of crosslinker (from 10% to 60% mol/mol) leading to a variation in the material stiffness (with Young's modulus in between 38 and 635 MPa). These different molecular compositions have been used to test the effect on the phenotype of neoplastic cells using the melanoma A375 cell line. Upon growth on LCNs, we found a reduction in cell proliferation and colony‐forming ability as well as an increase in cellular senescence. These effects were associated with a shift from a mesenchymal to a more epithelial phenotype, pointing to the occurrence of mesenchymal to epithelial transition. The former effects were more evident for cells grown on LCNs with a lower percentage of crosslinkers (LCN10 and LCN20), in line with the fact that tumor aggressiveness is linked to the increase in density and rigidity of the extracellular matrix. An important aspect is that all the observed effects on the cellular behavior have been obtained just thanks to the specific material‐cells interaction, so that any additional stimuli (e.g., specific growth factor) were not needed to obtain the observed effects. Further research will be focused to better elucidate the reasons behind this anti‐cancer effect, and to evaluate possible future applications of these materials in the clinical‐therapeutic field, such as in the field of post‐operative reconstruction involving prosthetic implants.

## 4 Experimental Section

### Materials Preparation

Liquid crystalline monomer 4‐methoxybenzoic acid 4‐(6‐acryloyloxy‐hexyloxy)phenyl ester (C6BP) was purchased from Synthon Chemicals. Liquid crystalline crosslinker 2‐methyl‐1,4‐phenylene bis(4‐(3‐(acryloyloxy)propoxy)benzoate) (RM257) was purchased from Wilshire Chemical Company. Radical photoinitiator (2‐Benzyl‐2‐(dimethylamino)−1‐[4‐(morpholinyl) phenyl)]−1‐butanone) (Irgacure 369) and dichloromethane were purchased from Merck. The polymeric films have been prepared as described in the Supporting Information.

### Scaffold Characterization

Polarized Optical Microscopy (POM) was performed with a Zeiss Axio Observer A1 microscope in cross‐polarized mode equipped with a Linkam PE120 hot stage and an Axio camera. Attenuated total reflection (ATR‐IR) spectra were recorded on a Spectrum Two Spectrometer by Perkin Elmer. Tensile tests were performed with a Tensometer 2020 (Alpha Technologies) equipped with custom‐made clamps^[^ ^15d^ ^]^ to secure the 50 µm‐thick specimens and with a 0.5 mm min^−1^ speed of the mobile upper crosshead. The tests were performed with a heated chamber to keep the temperature at 38 °C. The engineering stress (σ) and strain (ɛ) were calculated from the measured force and displacement data. Relevant mechanical properties, namely Young's modulus (E), maximum stress (σ_max_), and strain at break (ɛ_B_), were extracted from stress‐strain curves. For each formulation, at least six specimens were tested.

### Cells and Culture Conditions

The A375 melanoma cell line (CRL‐1619)^[^ ^28^ ^]^ which presents the BRAFV600E mutation in the BRAF gene, was obtained from ATCC (Manassas, VA, USA). Cells were maintained in Dulbecco's Modified Eagle's Medium (DMEM) supplemented with 10% heat‐inactivated fetal bovine serum (FBS), 2 mmol L^−1^ glutamine, 50 U/mL penicillin and 50 mg mL^−1^ streptomycin (complete medium) and kept in an incubator under standard conditions (37 °C with 5% CO_2_ and atmospheric O_2_). Once 80 % −90% confluence was reached, the DMEM was removed and the cells were washed with PBS (Dulbecco's Phosphate‐Buffered Saline), consisting of a buffered solution containing 80 g L^−1^ NaCl, 2.5 g L^−1^ KCl, Na_2_HPO_4_·2H_2_O 18 g L^−1^, KH_2_PO_4_ 2.2 g L^−1^. Subsequently, the cells were removed by incubation at 37 °C with Trypsin/EDTA 0.5 mg mL^−1^ in PBS and placed in a new plate at a 1:10 dilution. The number of viable cells was established by Trypan Blue staining.

Before each experiment, the LCNs were hydrated and sterilized through three washes for 5 minutes each on a shaker with ethanol diluted at 70% in sterile H_2_O, then another wash was carried out with PBS and dried in an oven. Subsequently, A375 cells were plated directly on plastic (7.5 × 10^4^ cells per well in 6‐well multiwell plates) for control (seeded on plastic) or onto LCNs (1.5 × 10^5^ cells per well in 6‐well multiwell plates). The different numbers of cells have been chosen to avoid confluency in the control samples at the end of the experiments and therefore possible masking of differences of proliferation rates compared to LCNs. Of note, after 24 hours, the percentages of cells attaching to the different LCNs, were comparable to those seeded on control plastic. After 24 hours, the LCNs were moved to other 6‐well multiwell plates to eliminate the cells attached to the bottom of the well. Pictures were taken after 24, 48, and 72 hours (six photos/well) to evaluate the number of cells. At 72 hours cells were detached from the LCNs by incubation at 37 °C with trypsin/EDTA for subsequent use in the experiments.

### Colony Formation Assay

Cells were recovered from 72‐hour‐culture on plastic or LCNs and seeded (500 cells/60 mm Petri dish) in complete medium and then allowed to grow for 7 days, as previously described.^[^ ^29^ ^]^ Subsequently, the medium was removed, the cells were washed twice with PBS and a crystal violet‐containing buffer (0.5% crystal violet, 30% ethanol, 3% formaldehyde) was added and left to act for 10 minutes. Colonies (i.e., more than 50 cells) were counted.

### Evaluation of Cellular Senescence by Senescence‐Associated βGal Staining

Cells were recovered from 72‐hour‐culture on plastic or LCNs and seeded (3.5 × 10^4^ cells per well in 6‐well multiwell plates) on culture plates for 72 hours. For the positive control for cellular senescence, 7×10^5^ cells were seeded and then treated with 200 µM H_2_O_2_ for 2 hours in an incubator at 37 °C. Cells were then allowed to grow in an incubator at 37 °C for 72 hours, and subsequently fixed with 2% formaldehyde for 10 minutes at room temperature. Cellular senescence was evaluated as previously reported.^[^ ^25b^ ^]^ Briefly, senescence‐associated (SA) βGal staining solution (X‐gal 1 mg mL^−1^, 40 mmol L^−1^ citric acid, 5 mmol L^−1^ C_6_FeK_4_N_6_, 5 mmol L^−1^ C_6_N_6_FeK_3_, 150 mmol L^−1^ NaCl and 2 mmol L^−1^ MgCl_2_; pH 5.9) was added for 16 hours (37 °C). Senescent cell quantification was performed by counting SA‐βGal–positive (blue) cells in 10 random images/well taken using a brightfield microscope. An average of 800 cells/conditions were counted.

### Cell Shape Analysis

After 72 hours of cultures on LCNs or control plastic, pictures were taken and major and minor cellular axes length was measured with ImageJ software. An average of 45 cells/conditions were counted.

### RNA Extraction, Reverse Transcription, and Real‐Time Quantitative PCR (qPCR)

Experiments were performed as previously described.^[^ ^30^ ^]^ Total RNA was isolated using TRIzol (Life Technologies). cDNA synthesis was performed using the ImProm‐II Reverse Transcription System, while quantitative PCR (qPCR) was performed using GoTaq qPCR Master Mix (Promega Corporation), following the manufacturer's protocol. Below, the study reports the sequence of the primers (forward FW and reverse RV) used:

‐E‐cadherin
• FW 5′‐CGG GAA TGC AGT TGA GGA TC‐3′
• RV 5′‐AGG ATG GTG TAA GCG ATG GC‐3′
‐N‐cadherin
• FW 5′‐GCA GAT CGG ACC GGA TAC TG‐3′
• RV 5′‐GTG GGA ATC GCA CGA ATG G −3′
‐ SNAIL
• FW 5′‐CCC AGT GCC TCG ACC ACT AT‐3′
• RV 5′‐ CCA GAT GAG CAT TGG CAG C‐3′

qPCR was performed using the CFX96 Touch real‐time PCR detection system (Bio‐Rad). mRNA expression was normalized to GAPDH and 18S mRNA. The relative expression of E‐cadherin, N‐cadherin, and Snail genes was calculated using the cycle threshold (CT) comparison method and the formula 2−ΔΔCt.^[^ ^31^ ^]^

### Statistics

Data represent mean ± standard deviation (SD) values calculated on at least three independent experiments. *P* values were calculated using Student's *t*‐test (two groups) or one‐way analysis of variance (ANOVA, multiple comparison using Tukey's honestly significant difference test). ≤0.05 was considered statistically significant.

## Conflict of Interest

The authors declare no conflict of interest.

## Supporting information



Supporting Information

## Data Availability

The data that support the findings of this study are available from the corresponding author upon reasonable request.
