# Supplementary material for: Liquid Crystalline Networks Hamper the Malignancy of Cancer Cells
Source: Adv Healthc Mater. 2025 Jan 19;14(7):2403607. doi: 10.1002/adhm.202403607 (PMC11912096; doi:10.1002/adhm.202403607)
Supplement: Supplementary file 1 — Supporting Information [file ADHM-14-0-s001.pdf]

ADVANCED HEALTHCARE MATERIALS

Supporting Information

for *Adv. Healthcare Mater.*, DOI 10.1002/adhm.202403607

Liquid Crystalline Networks Hamper the Malignancy of Cancer Cells

Daniele Martella, Ignazia Tusa, Alessandro Tubita, Alessia Negri, Marco Turriani, Marta Rojas-Rodríguez, Martina Salzano de Luna, Alessio Menconi, Camilla Parmeggiani and Elisabetta Rovida*

Supporting Information

Liquid Crystalline Networks hamper the malignancy of cancer cells

Daniele Martella, Ignazia Tusa, Alessandro Tubita, Alessia Negri, Marco Turriani, Marta Rojas-Rodríguez, Martina Salzano de Luna, Alessio Menconi, Camila Parmeggiani, Elisabetta Rovida*

Further information about material characterization and sample prepration are provided in the following sections.

1. Polarized optical microscope (POM) images of monomeric mixtures

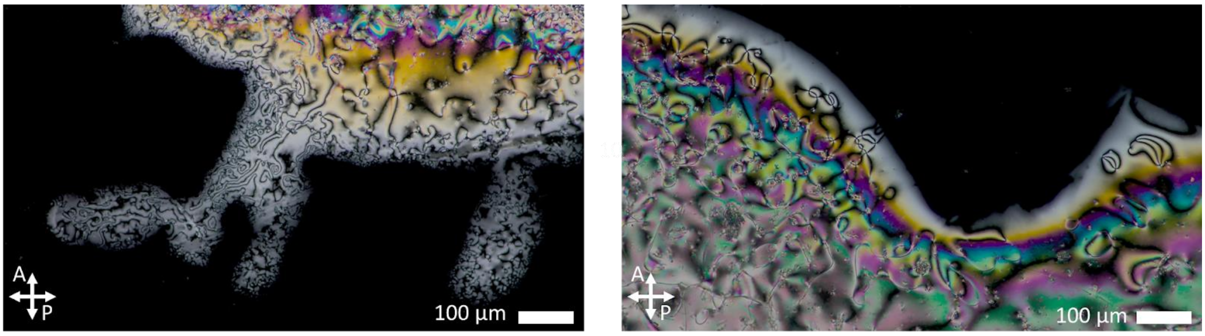

Figure S1. Polarized optical microscope (POM) images of monomeric mixtures. The scale label is 100 μm. The left image shows the monomeric mixture of LCN20 while the right image shows the monomeric mixture of LCN60. Both samples were heated to 75 °C and images were taken by cooling the sample to 45 °C. The mixtures clearly show Schlieren textures with two-fould and four-fould brushes typical of nematic mesophase.

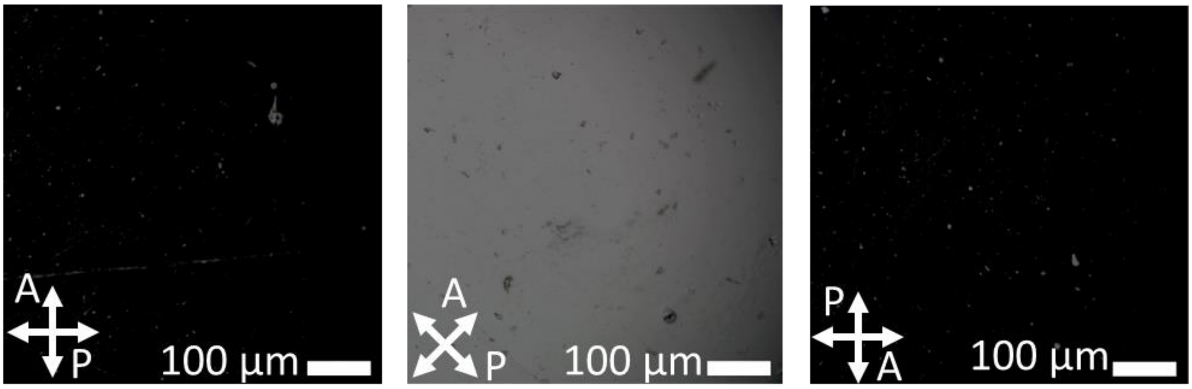

Figure S2. Polarized optical microscope (POM) images of LCN40 film. The scale label is 100 μm. The right and left images were obtained by orienting the director of the sample (direction of the rubbing in the preparation of the cells) parallel with one of the two crossed polarizers (A and P). The image in the middle was obtained by orienting the director of the sample at 45° with both the polarizers. The change in transmittance, due to the birefringence of the material, indicates a macroscopical alignment of the mesogens along a single direction.

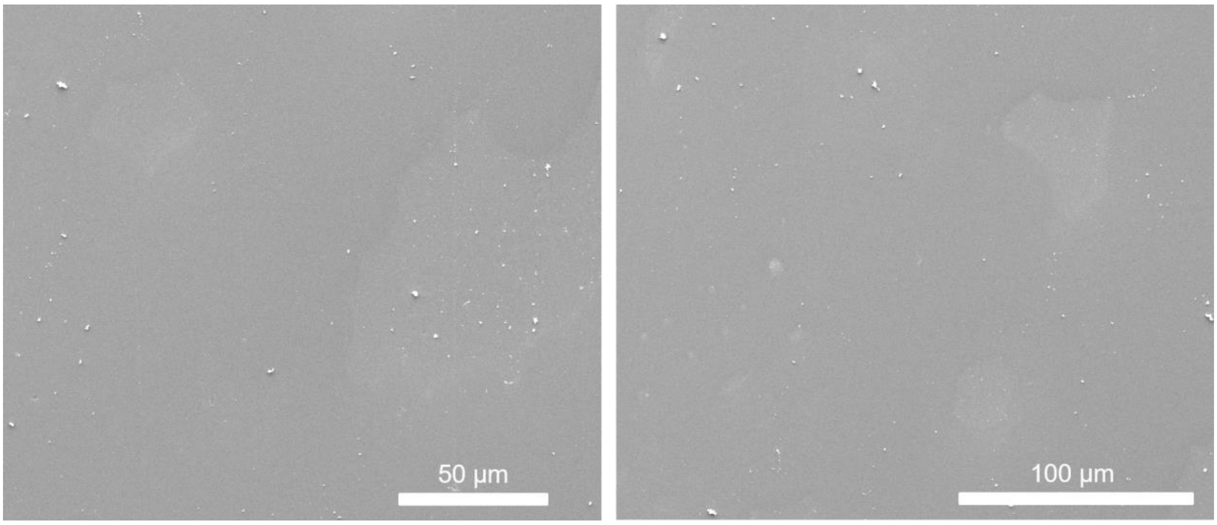

Figure S3. Scanning Electron Microscopy (SEM) images of the surface of a LCN20 film. Images have been recorded with a FlexSEM 1000 (Hitachi)

2. Procedure for scaffolds preparation

LCN films were prepared through photopolymerization of a monomeric mixture containing the mesogenic monomer C6BP (89-39% mol/mol), the mesogenic crosslinker RM257 (10-60% mol/mol) and the radical photoinitiator Irgacure 369 (1% mol/mol). Monomers were dissolved with some drops of dichloromethane and stirred at 65 °C for 20 minutes. The mixture was then dried under a vacuum, then heated at 65 °C and infiltrated by capillarity in a homemade polymerisation cell. The sample was cooled down to 45 °C to reach a nematic phase and then irradiated, firstly, for 10 min with a UV LED lamp (Thorlabs M385L2-C4, 385 nm, I = 1.8 mW cm^{-2}) and then, for a further 10 minutes at 65 °C. The cells were mechanically opened, and the film was removed by a blade.

The polymerization cells were composed of two glasses glued together and maintained separated by 50 µm-sized borosilicate glass spheres as spacers. The glasses were previously spin-coated (Pi-KEM LTD 6808, 5000 rpm 30 s) with a PVA solution (5% w/w in water) and rubbed unidirectionally with a velvet cloth to obtain a homogeneous planar alignment.
